# Supplementary material for: HuR controls glutaminase RNA metabolism
Source: Nat Commun. 2024 Jul 4;15:5620. doi: 10.1038/s41467-024-49874-x (PMC11224379; doi:10.1038/s41467-024-49874-x)
Supplement: Supplementary file 1 — Supplementary information [file 41467_2024_49874_MOESM1_ESM.pdf]

Supplementary Information

HuR controls glutaminase RNA metabolism

Douglas Adamoski[1,2], Larissa M dos Reis[1,2,3], Ana Carolina Paschoalini Mafra[1,2], Felipe Corrêa-da-Silva[2,3], Pedro Manoel Mendes de Moraes-Vieira[3], Ioana Berindan-Neagoe[4,5,6], George A. Calin[7,8], Sandra Martha Gomes Dias[1,*]

[1] Brazilian Biosciences National Laboratory (LNBio), Brazilian Center for Research in Energy and Materials (CNPEM), Campinas, Sao Paulo, Brazil

[2]Graduate Program in Genetics and Molecular Biology, Institute of Biology University of Campinas (UNICAMP), Campinas, Sao Paulo, Brazil

[3]Department of Genetics, Evolution, Microbiology, and Immunology, Laboratory of Immunometabolism, Institute of Biology, University of Campinas-UNICAMP, Campinas, SP, Brazil

[4]Research Center for Functional Genomics, Biomedicine and Translational Medicine, University of Medicine and Pharmacy "Iuliu-Hatieganu," Cluj-Napoca, Romania

[5]MedFuture Research Center for Advanced Medicine, University of Medicine and Pharmacy "Iuliu-Hatieganu," Cluj-Napoca, Romania

[6]Department of Functional Genomics and Experimental Pathology, The Oncology Institute "Prof. Dr. Ion Chiricuţă," Cluj-Napoca, Romania

[7]Department of Experimental Therapeutics, The University of Texas MD Anderson Cancer Center, 1515 Holcombe Blvd. Unit 1950, Houston, TX, USA.

[8]Center for RNA Inference and Non-Coding RNAs, The University of Texas MD Anderson Cancer Center, 1515 Holcombe Blvd. Unit 1950, Houston, TX, USA

*Corresponding author

Correspondence and requests for materials should be addressed to:
Sandra MG Dias, PhD
E-mail: sandra.dias@lnbio.cnpem.br

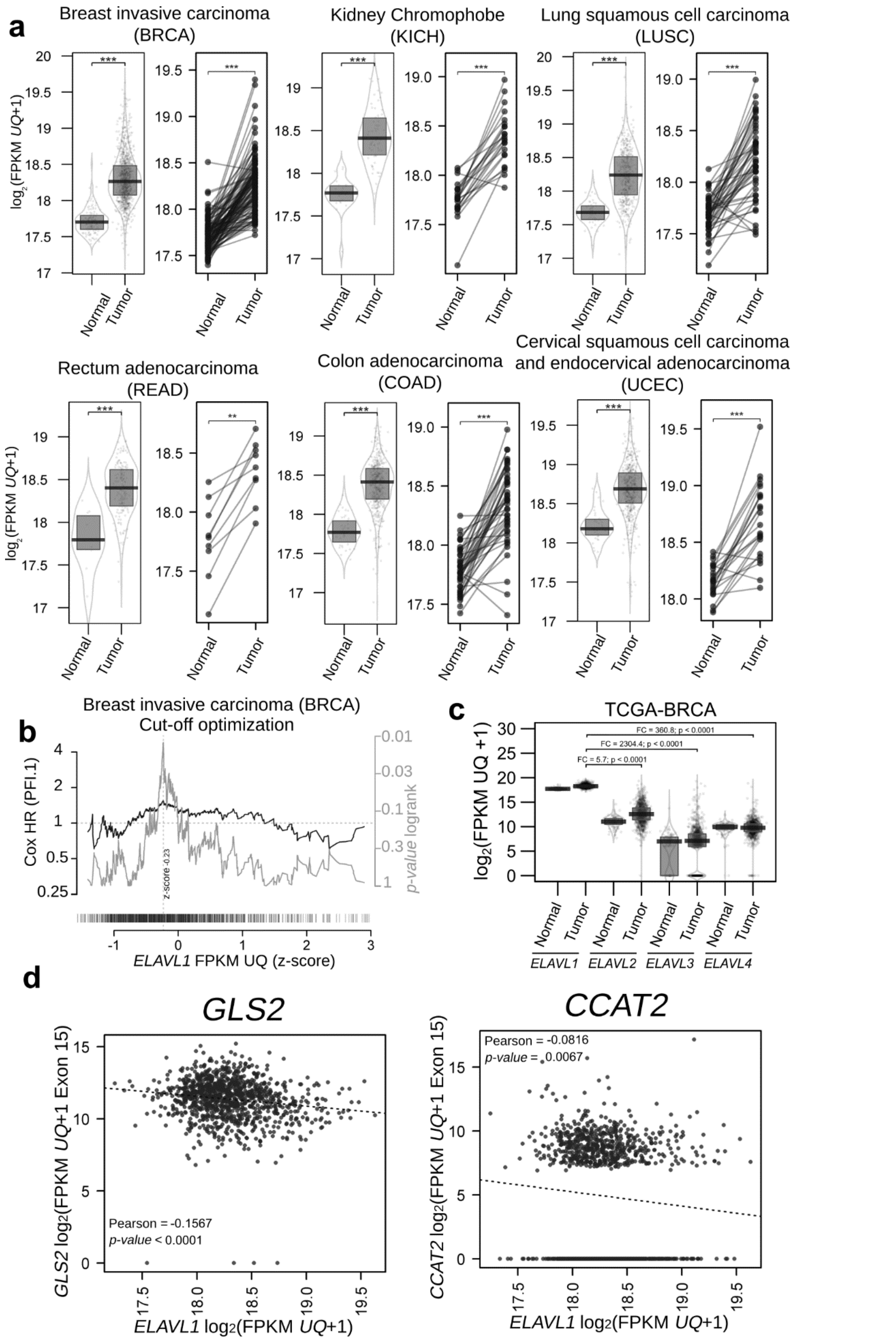

Suppl. Figure 1. *ELAVL1* expression is increased in multiple tumors compared to normal tissues and correlates positively with KGA and GAC mRNA levels in TCGA studies. (a) *ELAVL1* expression levels of normal adjacent tissues versus tumor (left) and paired normal-tumor

tissues (right) of breast cancer carcinoma, kidney chromophore, rectum and colon adenocarcinomas, lung and cervical squamous cell carcinomas. Data were obtained from the TCGA database. (**b**) Minimization of the p-value (gray line) of the Cox hazard ratio (black line) for disease-free survival using *ELAVL1* expression, splitting patients into a "low" or "high" expression level. (**c**) *ELAVL*-family expression among normal and tumor samples within TCGA-BRCA cohort including Fold-Changes (FC) and *p*-values from Tukey test after ANOVA. (**d**) *ELAVL1* correlated negatively with *GLS2* and *CCAT2* mRNA levels in the TCGA-BRCA cohort. Each vertical black line on the bottom represents a patient tumor. The vertical dashed line is the best-defined cutoff, and the horizontal dashed line indicates a Cox hazard ratio equal to one. Box plots represent the interquartile range; the vertical curve is the kernel density of the distribution, and the dark horizontal line denotes the mean. Statistical significance was derived from Welch's t-test (**a**, left plots), paired Student's t-test (**a**, right plots), log-rank test (**b**); Pearson correlation test (**c**); each dot represents an individual tumor sample. *$p < 0.05$, **$p < 0.01$, ***$p < 0.0001$.

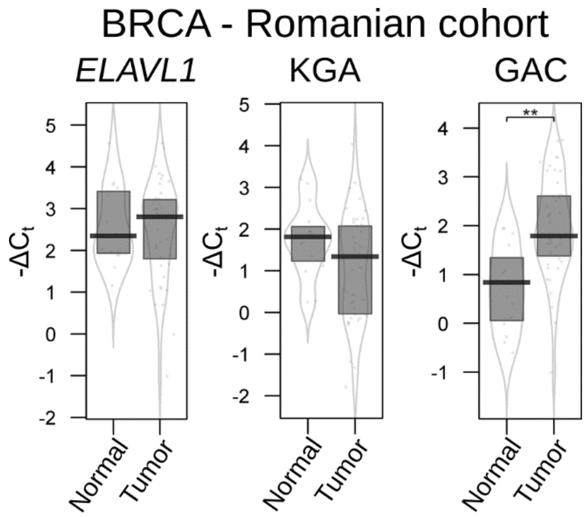

Suppl. Figure 2. The GAC isoform is overexpressed in a Romanian cohort of breast cancer patients. *ELAVL1*, KGA, and GAC mRNA levels assessed by qPCR in nonpaired normal breast tissue of the second cohort of breast tumors. Statistical significance derived from Welch's t-test; error bars are SEM; each point represents a patient. **p* < 0.05, ***p* < 0.01, ****p* < 0.0001.

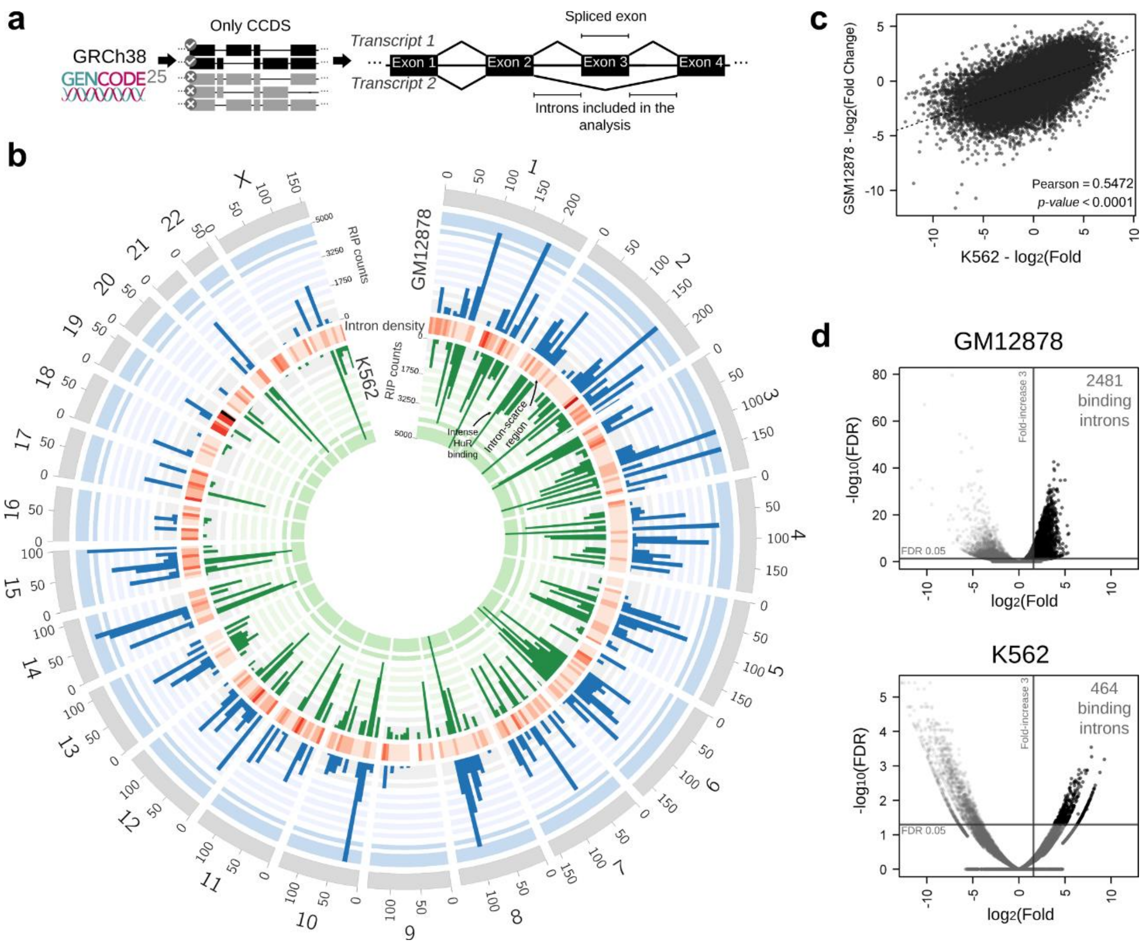

Suppl. Figure 3. Transcriptomics studies show that HuR binds to introns and potentially affects the alternative splicing of several genes. (**a**) Schematic representation of the annotation file used for quantification. GENCODE v25 gene structure annotation was used; transcripts not present in the CCDS database were removed. After this filtering, only introns neighboring nonshared exons were used in the evaluation. (**b**) Circos plot displaying the human chromosomes (out most circle), the density of evaluated introns per chromosome position (heatmap in the "intron density track"), and the ratio of IP:HuR over IP:IgG recovered reads (RIP counts) for each intronic region evaluated (bar plot). The external track is the GM12878 cell line, and the internal track is the K562 cell line. (**c**) Correlation plot between GM12878 and K562 cell lines fold change (IP: HuR/IP: IgG) per read. (**d**) Volcano plot for GM12878 (top) and K562 (bottom) for intron-binding analysis. The gray horizontal line represents an FDR of 0.05, and vertical lines represent a fold change cutoff of 3 (linear units) for unspecific binding (gray dots) and specific HuR binding (black dots). Statistical test derived from Pearson correlation (**c**) or DESeq[1] analysis (**d**).

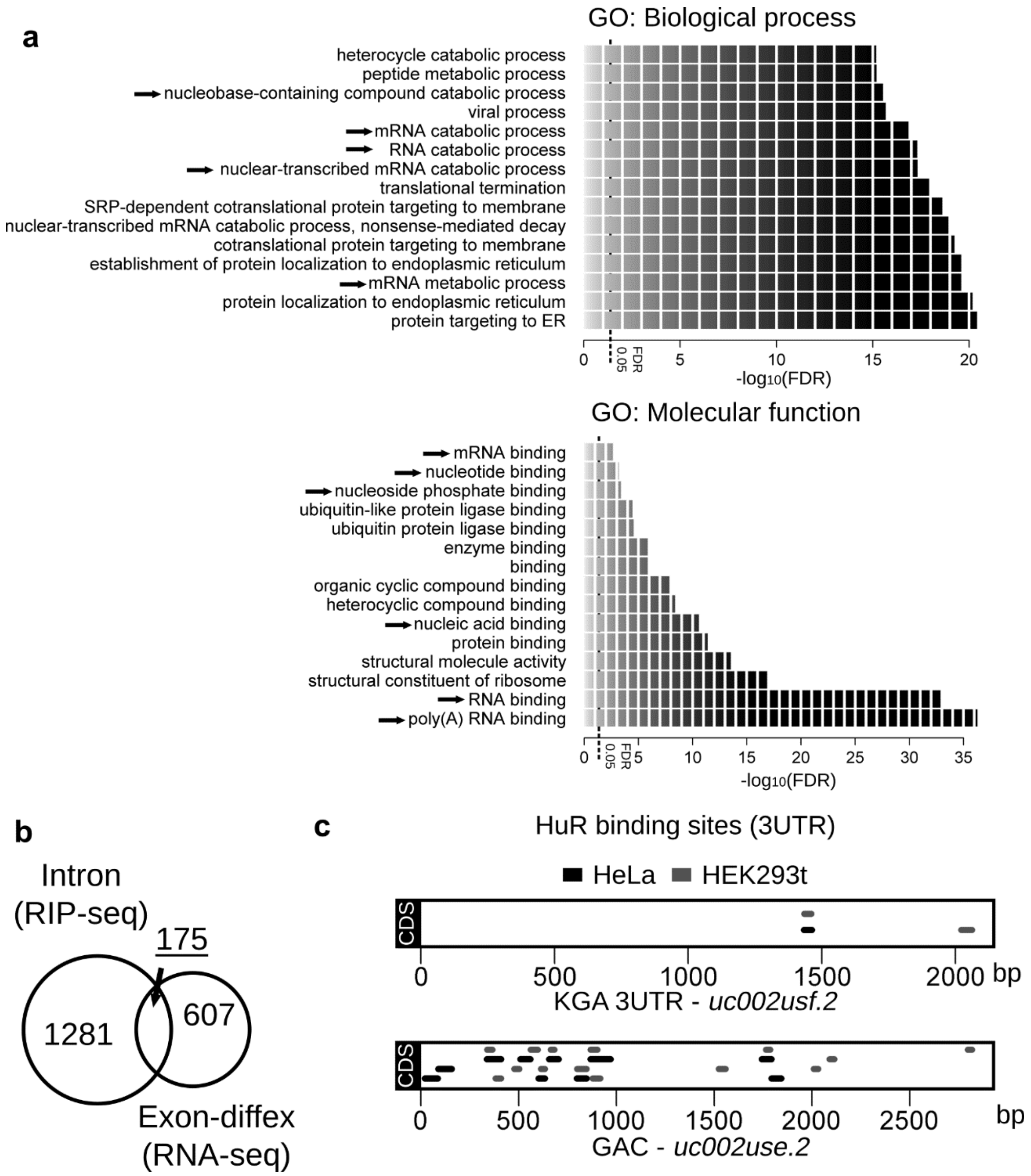

Suppl. Figure 4. Gene Ontology analysis indicates processes regulated by genes in which HuR binds to introns. (**a**) Gene ontology pathway enrichment analysis of the biological process (top) and molecular function (bottom) of genes whose exons presented differential expression after *ELAVL1* knockdown in HeLa cells. Arrows indicate RNA metabolism-related pathways. (**b**) The intersection between the list of genes obtained after the analysis of RNA-Seq from *ELAVL1* knockdown and control HeLa cells (exon differentially expressed) and the genes obtained from the RIP-Seq studies (introns bound to immunoprecipitated HuR from the GM12878 cells). (**c**) PAR-CLIP-Seq defined HuR binding regions on KGA's 3'-UTR (top) or GAC 3'-UTR (bottom) in HeLa and HEK293T cells, as obtained from the AURA[2] database.

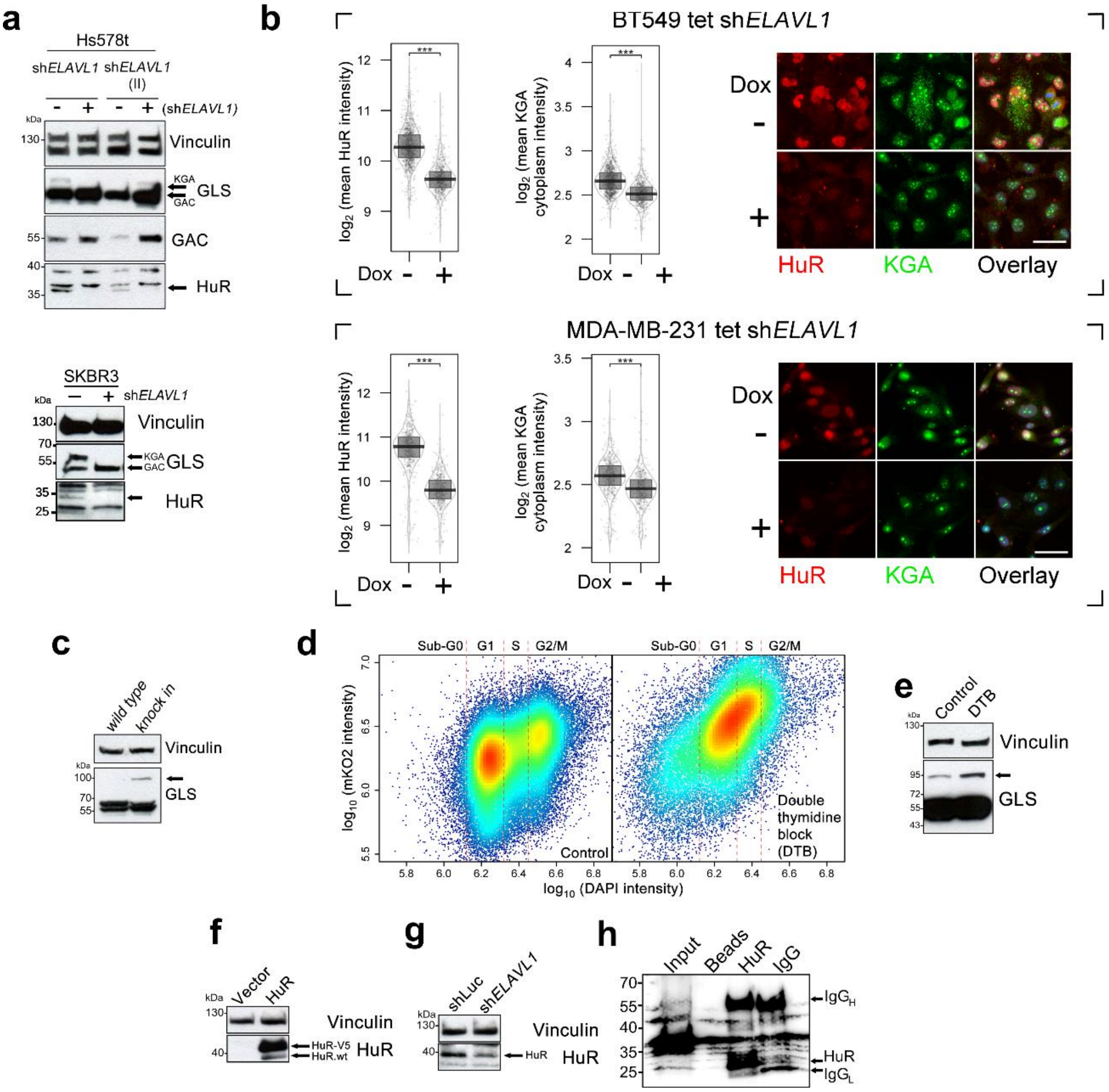

Suppl. Figure 5. *ELAVL1* knockdown affects KGA and GAC protein levels. (**a**) Doxycycline-induced *ELAVL1* knockdown of Hs578t and SKBR3 led to a marked decrease in KGA protein levels, accompanied by an increase in GAC. Arrows indicate KGA and GAC isoforms and HuR-specific bands. Short and long exposures are shown for glutaminase immunoblotting. (**b**) Immunofluorescence analysis of BT549 (above) and MDA-MB-231 (below) cell lines after *ELAVL1* doxycycline-induced knockdown. Quantification on the left and representative images on the right. (**c**) Demonstration by Western blot of size-increase from knock-in construct, arrow indicates KGA-mKO2 in HEK293T. (**d**) Cell cycle analysis of HEK293T knock-in cells before and after double thymidine block (DTB). DTB arrested cells in the G1-S phase, as expected; KGA levels (evaluated by mKO2 fluorescence) were also enhanced, as expected by findings from Colombo *et al*[3,4]. (**e**) Western blot for "d". The arrow indicates the shifted KGA-mKO2 isoform band. (**e**) Western blots for *ELAVL1* ectopic expression (**f**) and silencing (**g**) in HEK293T cells. Arrows indicate endogenous or ectopic HuR. (h) Immunoprecipitation of HuR in BT549 cell line using IgG control from same species as the HuR antibody (mouse). IgG_H = Immunoglobulin

heavy chain, IgG_L = Immunoglobulin light chain. Box plots represent the interquartile range; the vertical curve is the kernel density of the distribution, and the dark horizontal line denotes the mean. Each dot represents an individual cell. Statistical significance was derived from Welch's t-test. *$p < 0.05$, **$p < 0.01$, ***$p < 0.0001$.

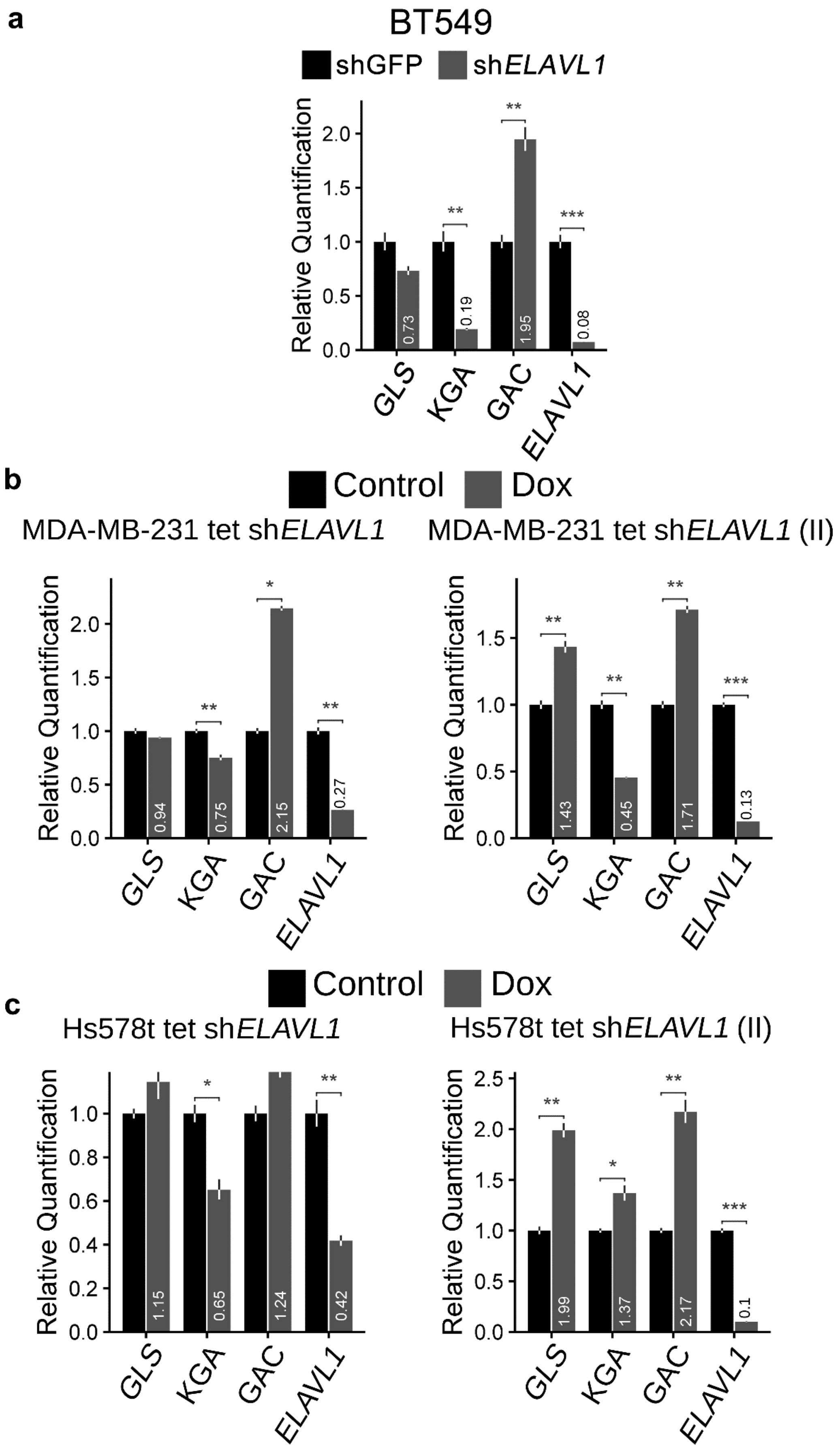
a
BT549
shGFP
shELAVL1
Relative Quantification
0.0
0.5
1.0
1.5
2.0
**
**
***
0.73
0.19
1.95
0.08
GLS
KGA
GAC
ELAVL1
b
Control
Dox
MDA-MB-231 tet shELAVL1
MDA-MB-231 tet shELAVL1 (II)
Relative Quantification
*
**
**
0.94
0.75
2.15
0.27
**
**
**
***
1.43
0.45
1.71
0.13
GLS
KGA
GAC
ELAVL1
c
Control
Dox
Hs578t tet shELAVL1
Hs578t tet shELAVL1 (II)
Relative Quantification
0.0
0.2
0.4
0.6
0.8
1.0
*
**
1.15
0.65
1.24
0.42
2.5
**
*
**
***
1.99
1.37
2.17
0.1
GLS
KGA
GAC
ELAVL1

Suppl. Figure 6. *ELAVL1* knockdown affects GAC and KGA mRNA levels. Quantitative relative mRNA quantification of *ELAVL1*, *GLS*, KGA, and GAC in constitutively-silenced BT549 (**a**), doxycycline-inducible MDA-MB-231 (**b**), and doxycycline-inducible Hs578t (**c**) cells after knockdown with sh*ELAVL1* I or sh*ELAVL1* II. Statistical significance was derived from Welch's t-test; each bar represents at least duplicates, and error bars are SEM. *$p < 0.05$, **$p < 0.01$, ***$p < 0.0001$.

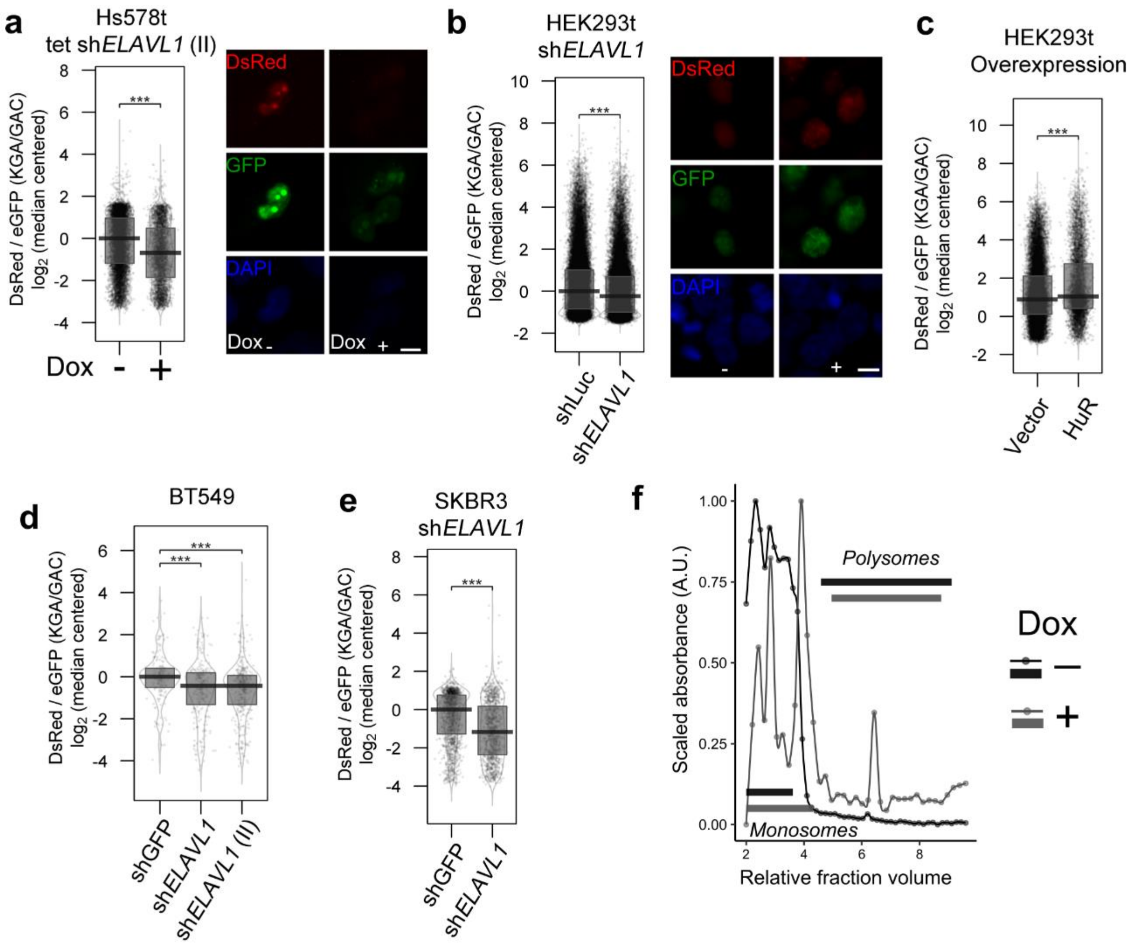

Suppl. Figure 7. *ELAVL1* knockdown affects *GLS* intron 14 removal as evaluated by a fluorescence sensor. *ELAVL1* doxycycline-induced knockdown decreased the DsRed/eGFP fluorescence ratio (an indicator of an increase in the preference for GAC over KGA mRNA during splicing events) in Hs578t (**a**, representative images on the right), HEK293T (**b**, above, representative images on the right), BT549 (**c**) and SKBR3 (**d**) cells. In B, *ELAVL1* ectopic expression was also evaluated (below). (**f**) Ultracentrifugation of BT549 cell lysates following dox-induction of ELAVL1 silencing using 10-50% step sucrose gradient. Fractioning performed manually, dots represent each real fraction, lines are a X-spline interpolation using ggplot2 and a spline_shape parameter of -0.5. Black or dark bars represent the pooled fractions used to qRT-PCR evaluations in Figure 04e. Box plots represent the interquartile range; the vertical curve is the kernel density of the distribution, and the dark horizontal line denotes the mean. Each dot represents an individual cell. Statistical significance was derived from Welch's t-test (**a**, **b**, and **d**) or ANOVA followed by Tukey's test (**c**). *$p < 0.05$, **$p < 0.01$, ***$p < 0.0001$.

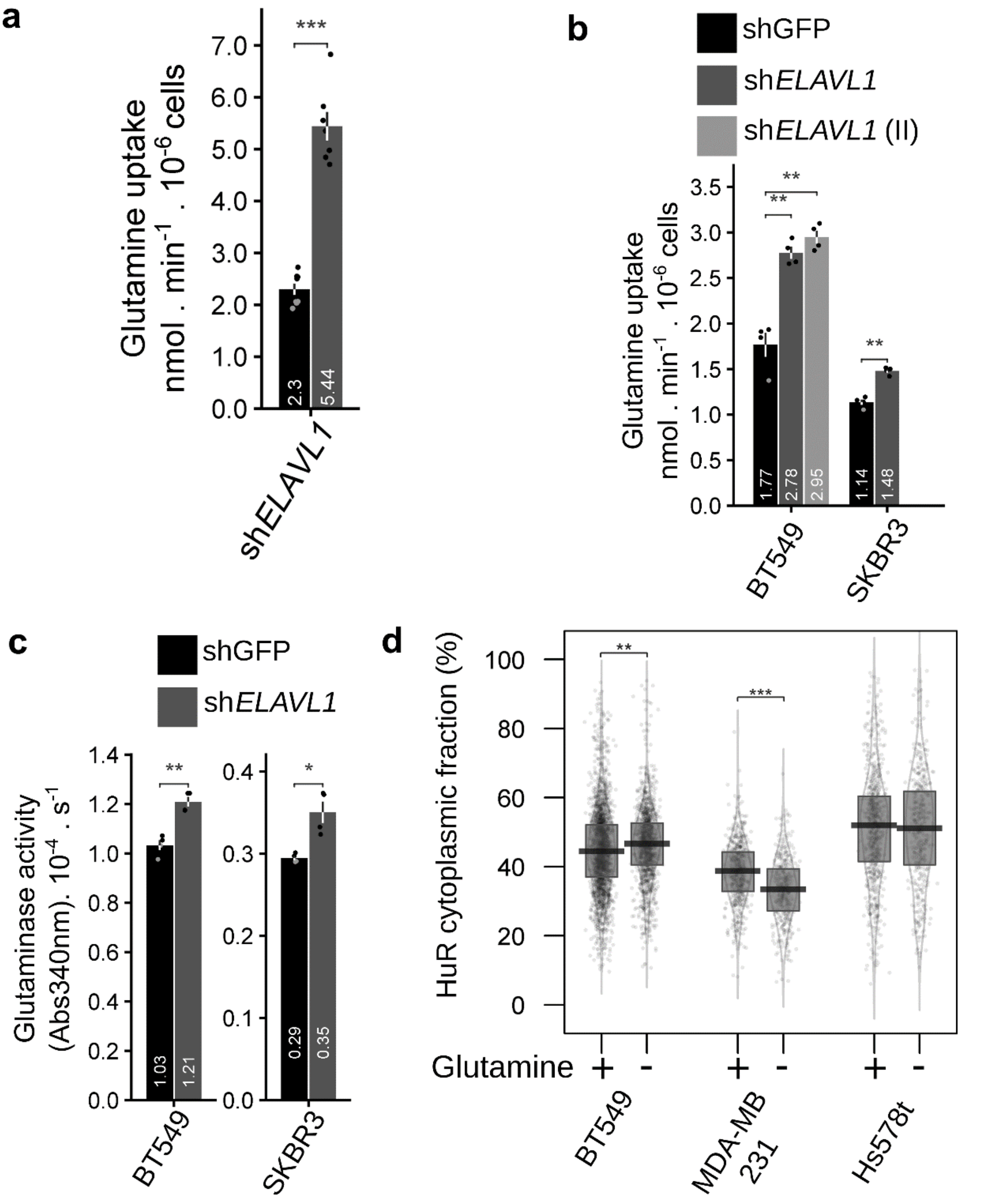

Suppl. Figure 8. *ELAVL1* knockdown affects glutamine metabolism in breast cancer cells. *ELAVL1* doxycycline-induced knockdown increased glutamine uptake in BT549 (**a**). *ELAVL1* stable knockdown increased glutamine uptake (**b**) and glutaminase activity (**c**) in BT549 and SKBR3 cells. (**d**) Immunofluorescence analysis of HuR staining in the nucleus and cytoplasm. Cytoplasmatic fraction calculated as a percentage of total cell staining in BT549, MDA-MB-231, and Hs578T cells in the presence or absence of glutamine in the media. Box plots represent the interquartile range; the vertical curve is the kernel density of the distribution, and the dark horizontal line denotes the mean. Each dot represents an individual cell. Statistical significance was derived from Welch's t-test (A, B – SKBR3, C and D) or ANOVA followed by Tukey's test (B – BT549), and error bars denote SEM. *$p < 0.05$, **$p < 0.01$, ***$p < 0.0001$.

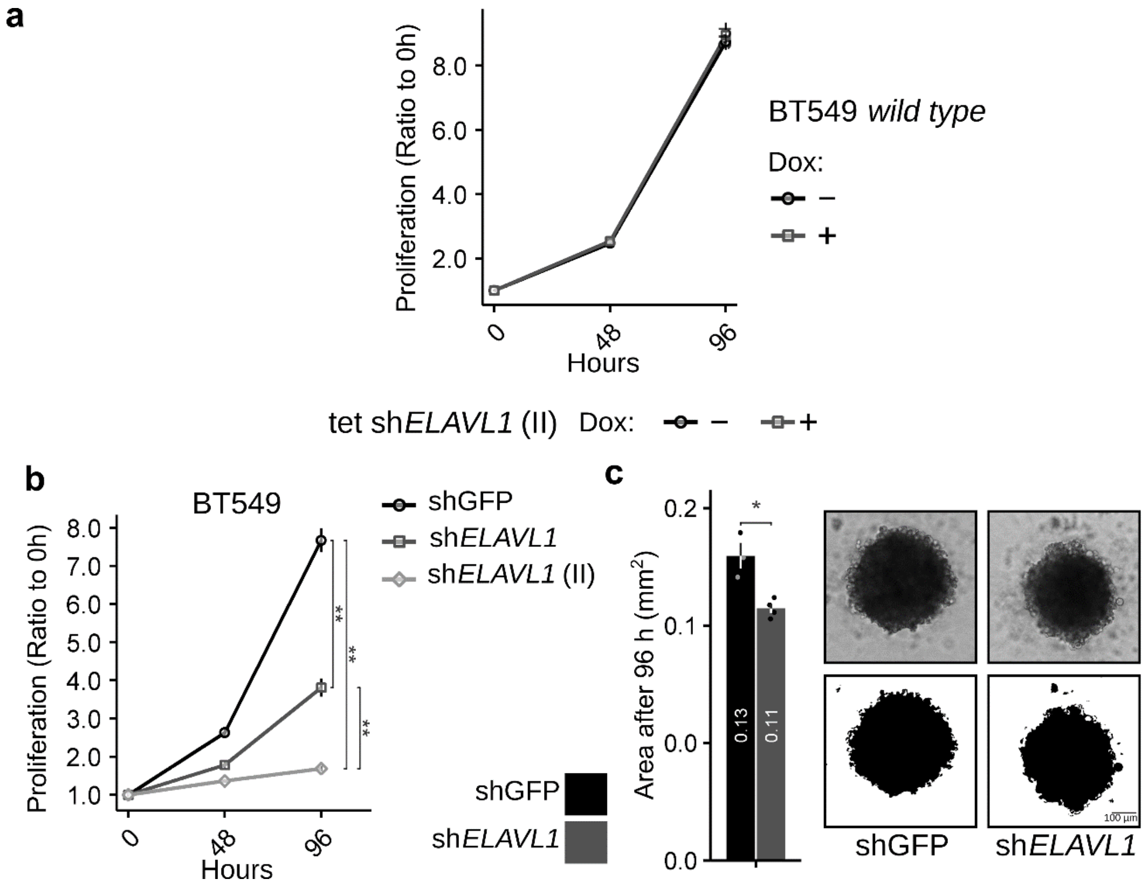

Suppl. Figure 9. *ELAVL1* knockdown affects breast cancer cell line proliferation. (**a**) Doxycycline, by itself, did not affect BT549 cell proliferation. Constitutive *ELAVL1* silencing using two shRNA sequences (sh*ELAVL1* and sh*ELAVL1* II) or only one (sh*ELAVL1*) decreased BT549 2D and 3D proliferation (**b** and **c**, respectively). Statistical significance was derived from Welch's t-test (A) or ANOVA followed by Tukey's test (**b**). Each dot represents an individual replicate; otherwise, n = 4 for proliferation assays; error bars are SEM. *$p < 0.05$, **$p < 0.01$, ***$p < 0.0001$.

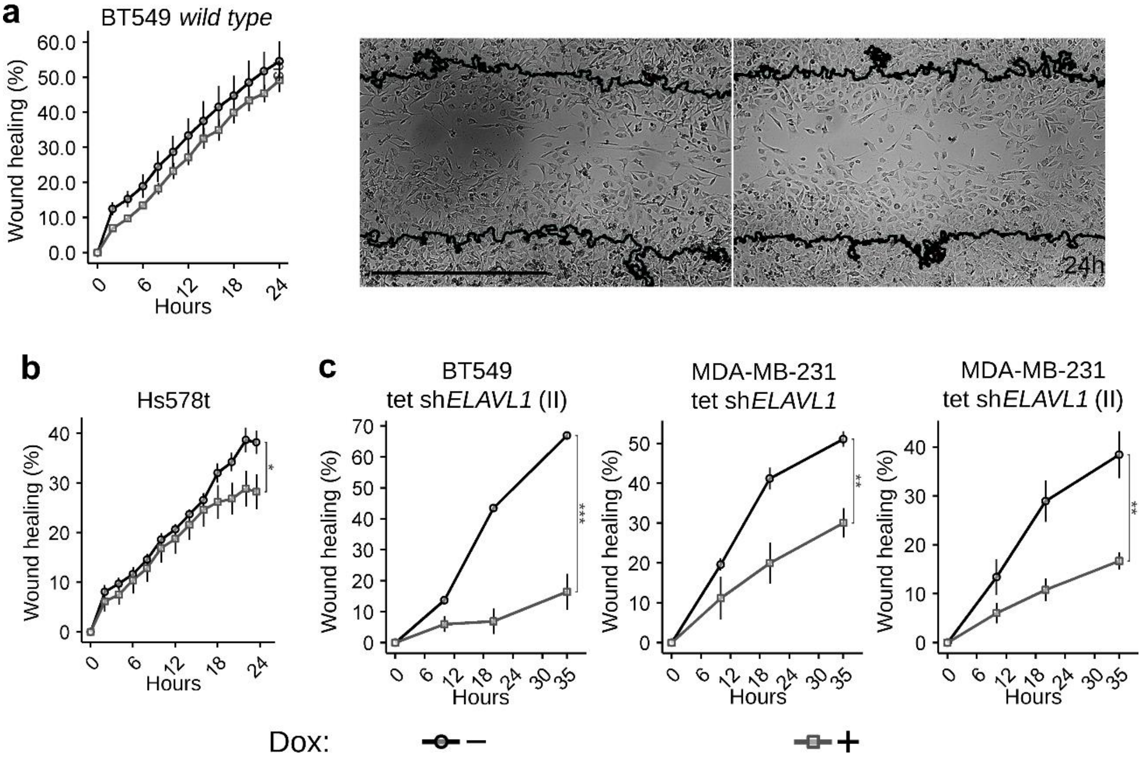

Suppl. Figure 10. *ELAVL1* knockdown affects breast cancer cell line migration and invasion. (**a**) Doxycycline, by itself, does not affect BT549 migration. (**b**) *ELAVL1* doxycycline-induced knockdown decreased Hs578t (sh*ELAVL1*) cell migration. *ELAVL1* doxycycline-induced knockdown decreased BT549 (sh*ELAVL1* II, **c**, left) and MDA-MB-231 (sh*ELAVL1* and sh*ELAVL1* II, **c**, middle, and right, respectively) cell invasion, as measured by the collagen matrix-overlaid scratch healing rate. Statistical significance was derived from Welch's t-test, and each dot represents an individual replicate; otherwise, n = 8 for migration assays; error bars are SEM. *$p < 0.05$, **$p < 0.01$, ***$p < 0.0001$.

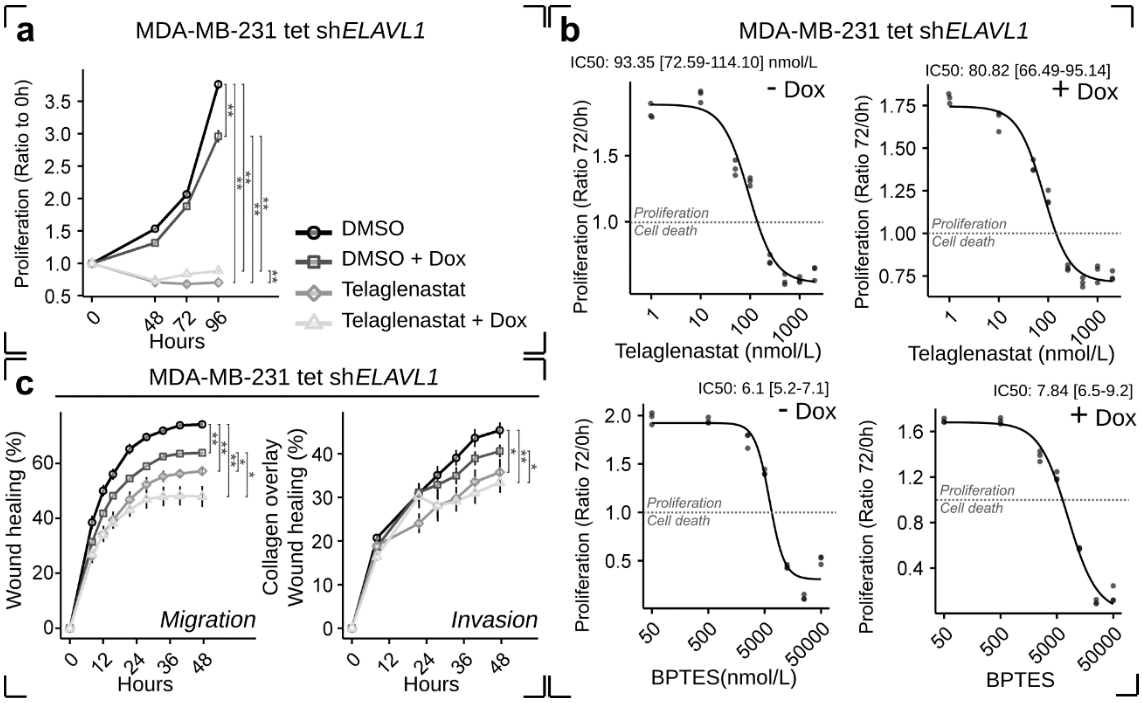

Suppl. Figure 11. *ELAVL1* knockdown sensitizes breast cancer cells to glutaminase inhibition. (**a**) Doxycycline-induced *ELAVL1* silencing in MDA-MB-231 cells combined with GLS inhibition with telaglenastat (CB-839) decreased cell proliferation by 19% and lead to cell death, respectively, compared to that in control cells (DMSO without doxycycline). (**b**) Growth response of MDA-MB-231 to increasing amounts of telaglenastat (above) or BPTES (bellow) with *ELAVL1* knockdown induced (-Dox, left) or not (+Dox, right). The IC50 value and 95% confidence interval (CI) are presented above the graphs. The gray dashed line indicates that there was a reduction in the final cell number compared to the number of seeded cells, denoting cell death. (**c**) Doxycycline-induced *ELAVL1* silencing of MDA-MB-231 cells combined with GLS inhibition and telaglenastat decreased cell migration (left) and invasion (right), compared to control cells (DMSO - Dox). Statistical significance was derived from Welch's t-test; each dot represents an individual replicate; otherwise, n = 4 for proliferation assays and n = 8 for migration/invasion assays; error bars are SEM. *p < 0.05, **p < 0.01, ***p < 0.0001.

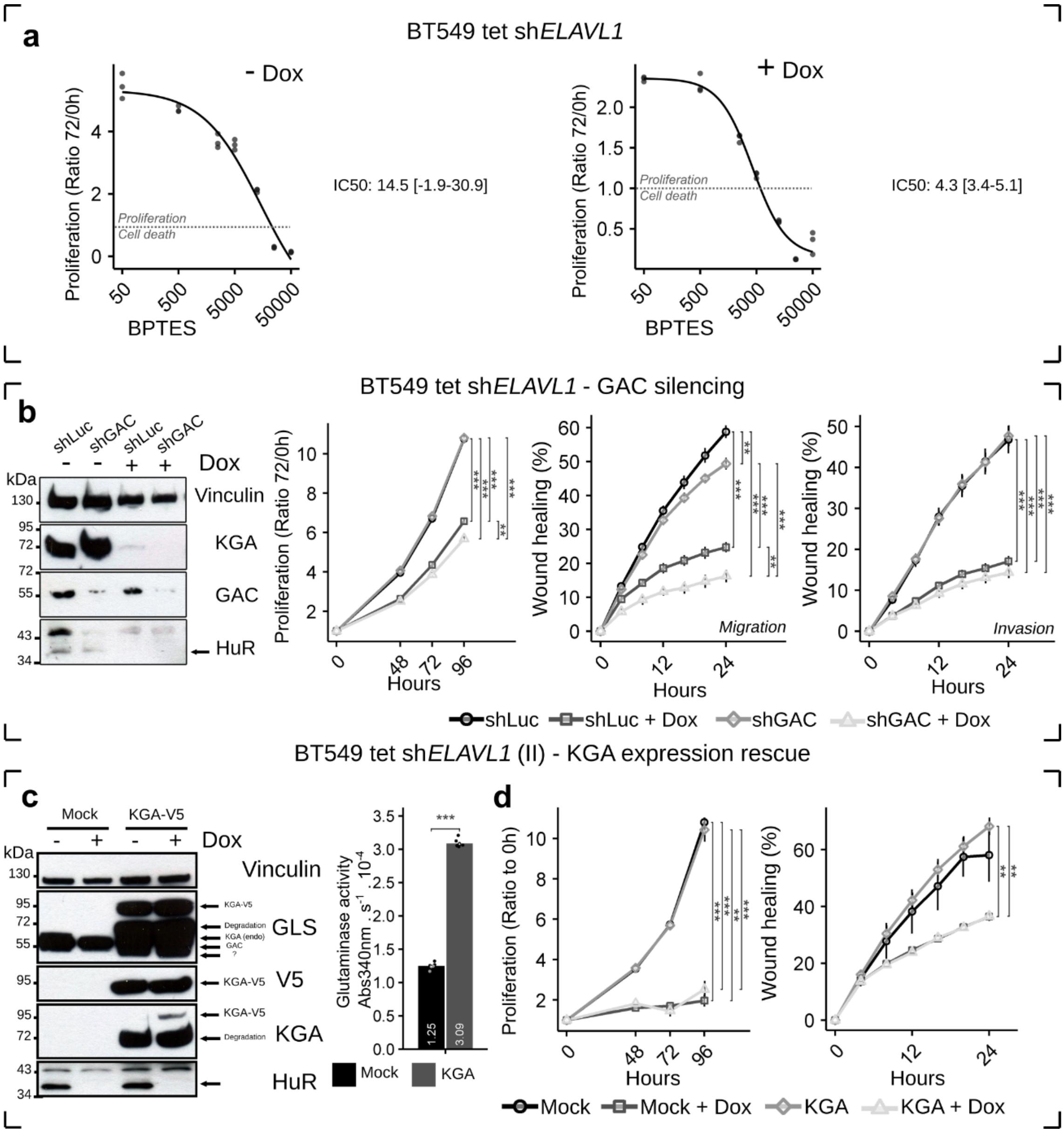

Suppl. Figure 12. *ELAVL1* knockdown sensitizes cells to glutaminase inhibition with BPTES, but neither GAC knockdown nor KGA ectopic expression affects the growth response. (**a**) Proliferation curves of BT549 cells after doxycycline-induced *ELAVL1* knockdown and treatment with increasing BPTES concentrations; under *ELAVL1* knockdown, cell death was detected at lower doses of BPTES compared to the control (-Dox). GAC knockdown (**b**, western blot on the left) slightly further decreased cell growth, migration, and collagen-overlaid invasion after *ELAVL1* knockdown (**b**, graphs on the right). KGA ectopic expression (**c**, western blot on the left) increased glutaminase activity (**c**, on the right) but did not affect proliferation and wound healing curves either with or without *ELAVL1* knockdown (**d**). Statistical significance was derived from Welch's t-test (**c**) or ANOVA followed by Tukey's test (**b** and **d**). Each dot represents an individual replicate; otherwise, n = 4 for proliferation assays and n = 8 for migration assays; error bars are SEM. *$p < 0.05$, **$p < 0.01$, ***$p < 0.0001$.

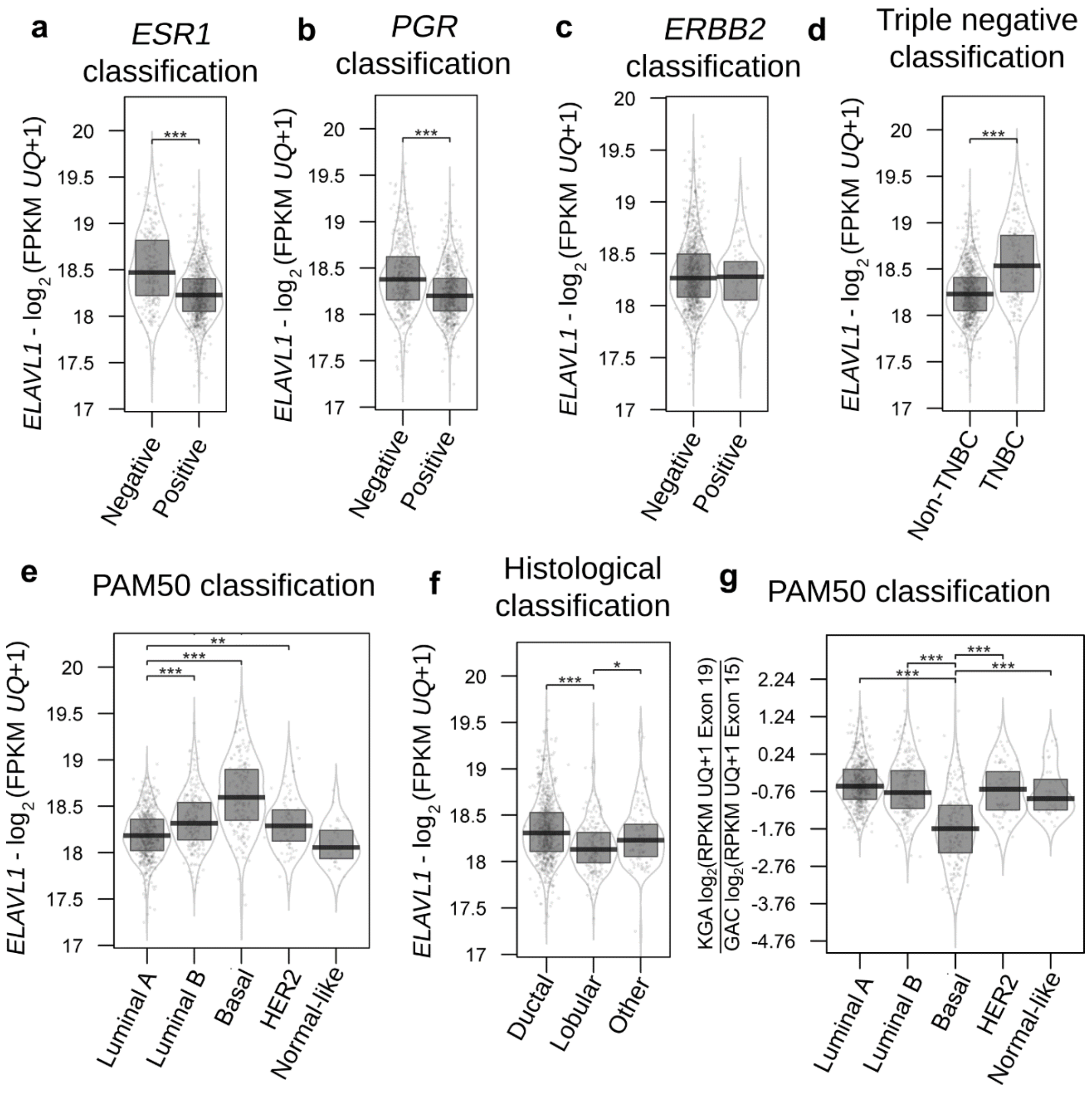

Suppl. Figure 13. ***ELAVL1*** **expression is increased in TCGA cohort breast cancer subtypes.** *ELAVL1* mRNA expression in accordingly mRNA expression of estrogen receptor (**a**), progesterone receptor (**b**), HER2/*ERBB2* (**c**) and overall triple negative breast cancer status (**d**). *ELAVL1* mRNA expression levels in PAM50 breast cancer subtyping (**e**) and histological classification (**f**). Ratio KGA/GAC mRNA expression levels in PAM50 breast cancer subtyping (**g**). Box plots represent the interquartile range; the vertical curve is the kernel density of the distribution, and the dark horizontal line denotes the mean. Statistical significance was derived from Welch's t-test; each dot represents an individual tumor sample. *$p < 0.05$, **$p < 0.01$, ***$p < 0.0001$.

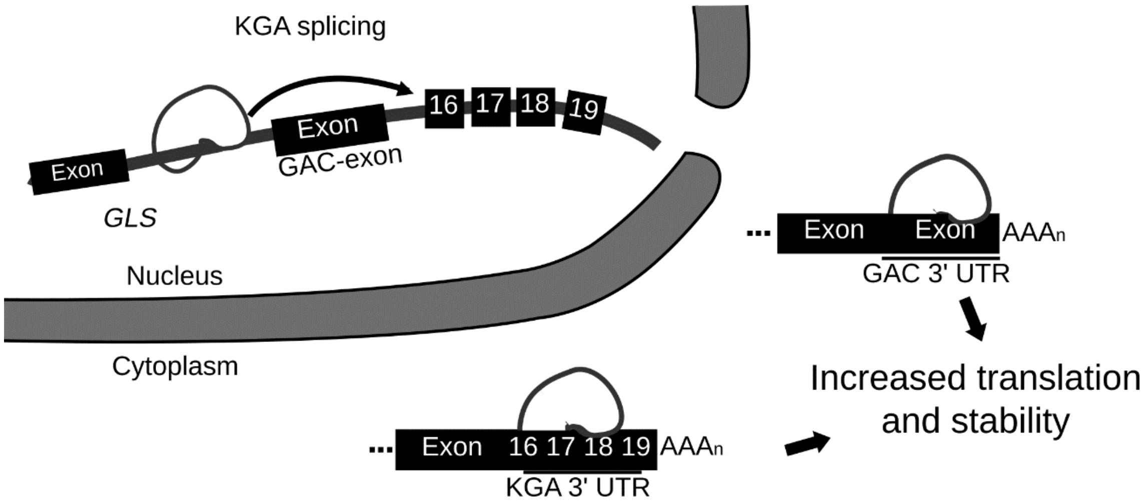

Suppl. Figure 14. Scheme illustrating all the GLS RNA metabolic processes controlled by HuR. This model illustrates the proposed splicing mechanism performed by HuR (which binds to *GLS* intron 14 and skips exon 15, termed the GAC exon) and the subsequent cytoplasmic fate of the *GLS* gene products. Both gene products are upregulated by HuR through increased translation and stability.

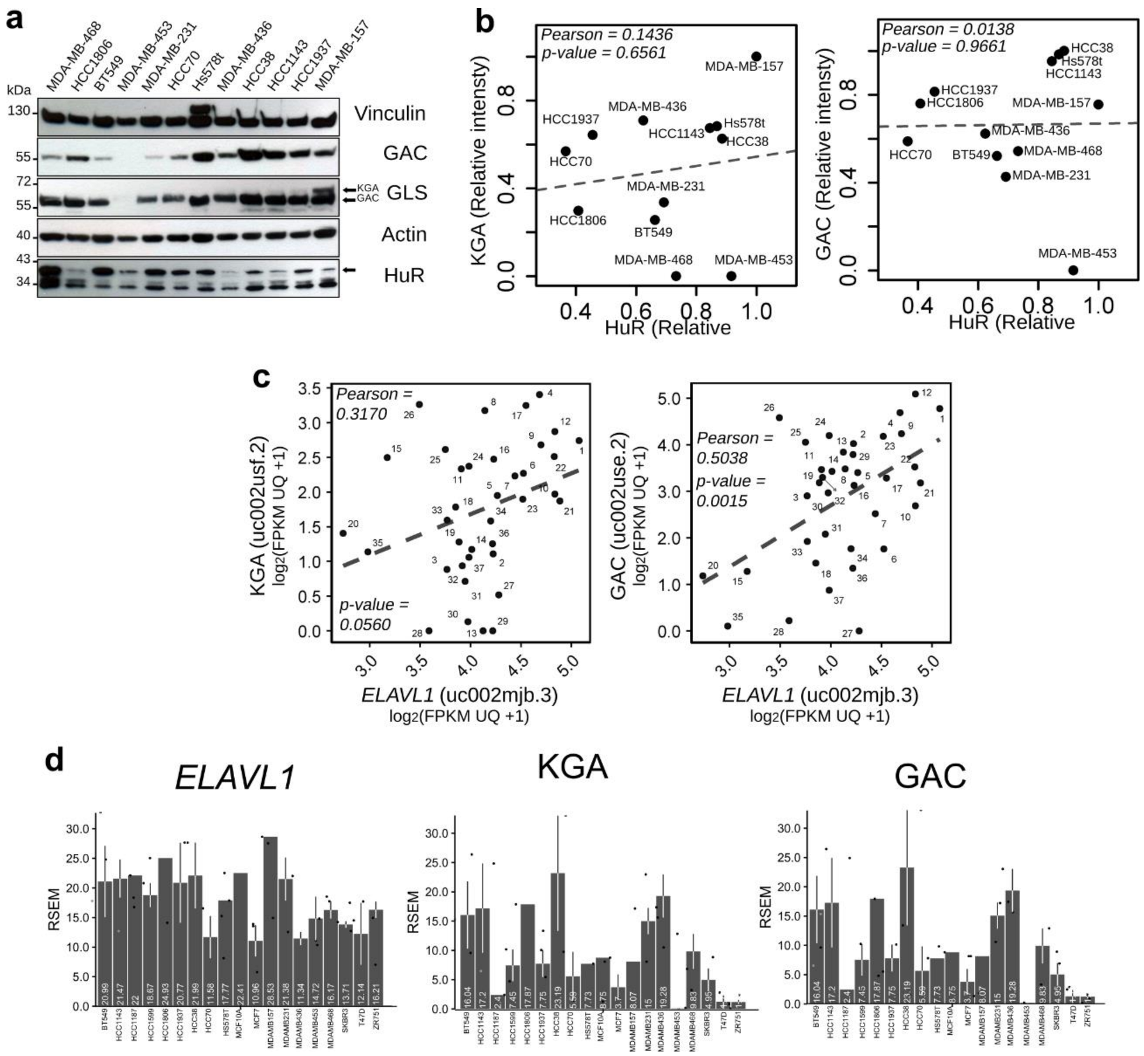

Suppl. Figure 15. HuR, GAC and KGA are ubiquitously expressed in several breast cancer cell lines. (**a**) Immunoblotting of twelve breast cancer cell lines using a GLS antibody that recognizes both GAC and KGA proteins, as well as a GAC exclusive antibody and an HuR antibody; vinculin and actin were used as loading controls. The indicated bands were quantified by densitometry, and the KGA x HuR (B, left) and GAC x HuR (**b**, right) band intensity values were correlated. (**c**) mRNA level correlation between *ELAVL1* and KGA (left) or GAC (right) using RNA-Seq data obtained from previous studies[5–7]. The cell lines evaluated were as follows: 1 - BT549 (Daemen), 2 - BT549 (Quintero), 3 - BT549 (Varley), 4 - HCC1143 (Daemen), 5 - HCC1143 (Varley), 6 - HCC1187 (Varley), 7 - HCC1599 (Daemen), 8 - HCC1599 (Varley), 9 - HCC1806 (Daemen), 10 - HCC1937 (Daemen), 11 - HCC1937 (Varley), 12 - HCC38 (Daemen), 13 - HCC38 (Varley), 14 - HCC70 (Daemen), 15 - HCC70 (Varley), 16 - HS578T (Daemen), 17 - MCF10A (Daemen), 18 - MCF7 (Daemen), 19 - MCF7 (Quintero), 20 - MCF7 (Varley), 21 - MDAMB157 (Daemen), 22 - MDAMB231 (Daemen), 23 - MDAMB231 (Quintero), 24 - MDAMB231 (Varley), 25 - MDAMB436 (Quintero), 26 - MDAMB436 (Varley), 27 - MDAMB453 (Daemen), 28 - MDAMB453 (Varley), 29 - MDAMB468 (Quintero), 30 -

MDAMB468 (Varley), 31 - SKBR3 (Daemen), 32 - SKBR3 (Quintero), 33 - SKBR3 (Varley), 34 - T47D (Daemen), 35 - T47D (Varley), 36 - ZR751 (Daemen), and 37 - ZR751 (Varley). All correlations and *p*-values were obtained from Pearson's correlation test. (**d**) Expression levels from “c” represented as barplots.

Supplementary references

1. Love, M. I., Huber, W. & Anders, S. Moderated estimation of fold change and dispersion for RNA-seq data with DESeq2. *Genome Biol* **15**, 550 (2014).

2. Dassi, E. *et al.* AURA 2: Empowering discovery of post-transcriptional networks. *Translation* **2**, e27738 (2014).

3. Colombo, S. L. *et al.* Molecular basis for the differential use of glucose and glutamine in cell proliferation as revealed by synchronized HeLa cells. *Proc Natl Acad Sci U S A* **108**, 21069–74 (2011).

4. Colombo, S. L. *et al.* Anaphase-promoting complex/cyclosome-Cdh1 coordinates glycolysis and glutaminolysis with transition to S phase in human T lymphocytes. *Proceedings of the National Academy of Sciences* **107**, 18868–18873 (2010).

5. Varley, K. E. *et al.* Recurrent read-through fusion transcripts in breast cancer. *Breast Cancer Research and Treatment* **146**, 287–297 (2014).

6. Daemen, A. *et al.* Modeling precision treatment of breast cancer. *Genome biology* **14**, R110 (2013).

7. Quintero, M. *et al.* Guanylate-binding protein-1 is a potential new therapeutic target for triple-negative breast cancer. *BMC Cancer* **17**, 727 (2017).
