# Supplementary material for: HuR controls glutaminase RNA metabolism
Source: Nat Commun. 2024 Jul 4;15:5620. doi: 10.1038/s41467-024-49874-x (PMC11224379; doi:10.1038/s41467-024-49874-x)
Supplement: Supplementary file 2 — Description of Additional Supplementary Files [file 41467_2024_49874_MOESM2_ESM.pdf]

Supplementary Data 1
Differential Expression Analysis of *ELAVL1* in Normal vs. Tumor Samples from the TCGA Database

Supplementary Data 2
Correlation of Intronic HuR Binding Sites (Identified by RIP-Seq) and Differentially Expressed Exons upon *ELAVL1* Knockdown (RNA-seq Data): Listing 175 Genes Potentially Regulated by HuR via Alternative Splicing

Supplementary Data 3
Identification and statistical test of a Putative HuR Binding Site within *GLS'* Intron 14

Supplementary Data 4
Comparative Expression Levels of Genes in Glucose Metabolism, TCA Cycle, Fatty Acid Synthesis, Beta-Oxidation, and Urea Cycle between High and Low *ELAVL1* Expressions in TCGA-BRCA Database
