# Supplementary material for: HuR controls glutaminase RNA metabolism
Source: Nat Commun. 2024 Jul 4;15:5620. doi: 10.1038/s41467-024-49874-x (PMC11224379; doi:10.1038/s41467-024-49874-x)
Supplement: Supplementary file 3 — Reporting Summary [file 41467_2024_49874_MOESM3_ESM.pdf]

nature portfolio

Corresponding author(s): Sandra Martha Gomes Dias

Last updated by author(s): Mar 3, 2024

Reporting Summary

Nature Portfolio wishes to improve the reproducibility of the work that we publish. This form provides structure for consistency and transparency in reporting. For further information on Nature Portfolio policies, see our Editorial Policies and the Editorial Policy Checklist.

Statistics

For all statistical analyses, confirm that the following items are present in the figure legend, table legend, main text, or Methods section.

n/a	Confirmed	
☐	☒	The exact sample size (*n*) for each experimental group/condition, given as a discrete number and unit of measurement
☐	☒	A statement on whether measurements were taken from distinct samples or whether the same sample was measured repeatedly
☐	☒	The statistical test(s) used AND whether they are one- or two-sided<br>*Only common tests should be described solely by name; describe more complex techniques in the Methods section.*
☐	☒	A description of all covariates tested
☐	☒	A description of any assumptions or corrections, such as tests of normality and adjustment for multiple comparisons
☐	☒	A full description of the statistical parameters including central tendency (e.g. means) or other basic estimates (e.g. regression coefficient) AND variation (e.g. standard deviation) or associated estimates of uncertainty (e.g. confidence intervals)
☐	☒	For null hypothesis testing, the test statistic (e.g. *F*, *t*, *r*) with confidence intervals, effect sizes, degrees of freedom and *P* value noted<br>*Give P values as exact values whenever suitable.*
☒	☐	For Bayesian analysis, information on the choice of priors and Markov chain Monte Carlo settings
☒	☐	For hierarchical and complex designs, identification of the appropriate level for tests and full reporting of outcomes
☐	☒	Estimates of effect sizes (e.g. Cohen's *d*, Pearson's *r*), indicating how they were calculated

Our web collection on statistics for biologists contains articles on many of the points above.

Software and code

Policy information about availability of computer code

Data collection

TCGA data were obtained from the Genomic Data Commons portal. The gene-level expression was defined in upper-quantile FPKM units, isoform-level expression was defined in RPKM units of UCSC isoforms in legacy TCGA pipeline. . RIP-Seq data was obtained from ENCODE project (SRR504455-6 and SRR504447-8 for GM12878; SRR504459-60 and SRR504453 for K562), RNA-Seq data for ELAVL1 silencing in HeLa cells were obtained from Lebedeva et al.

Data analysis

For body-map representation, data was plotted using QGIS and R-packages maptools, ggplot2, and gpclib. For progression-free interval p-value minimization, R-packages survival and survminer were used. Briefly, all possible cutoffs dividing patients into two groups were evaluated by log-rank tests, and the division with the smallest p-value was used. Quality was evaluated with FastQC; sequence trimming was performed by Skewer following published guidelines, sequences were aligned to GRCh38 using STAR (2-pass mode). For RIP-Seq analysis, each GENCODE (v25) transcript without CCDS entry was removed, retaining the only gene with two or more transcripts (exception for genes with transcripts varying only for transcriptional start sites, which were removed from the analysis). This new annotation was used to detect introns around exons varying among the isoforms, and HTSeq was used to count the aligned reads over those introns. Since no replicates were available, reads were subsampled in a bootstrap-like approach, as previous proposed, and counts used for DESeq2 analysis. For RNA-Seq analysis of HeLa siELAVL1, original GENCODE annotation was used, and DEXSeq evaluated differential exon usage, with the same bootstrap-like approach proposed. Gene ontology enrichment was evaluated using Go.db and goseq. Aligned PAR-CLIP-Seq data was analyzed with IGV. Evolutionary conservation of intron 14 was described with VISTA Browser and previously obtained sequences. RNA-Seq analysis of TNBC cell lines was performed as described previously.

For manuscripts utilizing custom algorithms or software that are central to the research but not yet described in published literature, software must be made available to editors and reviewers. We strongly encourage code deposition in a community repository (e.g. GitHub). See the Nature Portfolio guidelines for submitting code & software for further information.

Data

Policy information about availability of data

All manuscripts must include a data availability statement. This statement should provide the following information, where applicable:

- Accession codes, unique identifiers, or web links for publicly available datasets
- A description of any restrictions on data availability
- For clinical datasets or third party data, please ensure that the statement adheres to our policy

The authors declare that the data supporting the findings of this study are available within the paper and its supplementary information files. The external data that support the bioinformatic findings of this study are based upon data generated by the TCGA Research Network: http://cancergenome. nih.gov/ or other studies deposited in NCBI's SRA database.

Research involving human participants, their data, or biological material

Policy information about studies with human participants or human data. See also policy information about sex, gender (identity/presentation), and sexual orientation and race, ethnicity and racism.

Reporting on sex and gender	n/a
Reporting on race, ethnicity, or other socially relevant groupings	n/a
Population characteristics	n/a
Recruitment	n/a
Ethics oversight	n/a

Note that full information on the approval of the study protocol must also be provided in the manuscript.

Field-specific reporting

Please select the one below that is the best fit for your research. If you are not sure, read the appropriate sections before making your selection.

☒ Life sciences ☐ Behavioural & social sciences ☐ Ecological, evolutionary & environmental sciences

For a reference copy of the document with all sections, see nature.com/documents/nr-reporting-summary-flat.pdf

Life sciences study design

All studies must disclose on these points even when the disclosure is negative.

Sample size	All biological experiments were performed in triplicates for each cell line.
Data exclusions	No data were excluded
Replication	Replication was performed at least two times for each experiment
Randomization	No randomization was needed to the type of experiments performed
Blinding	No blinding were performed

Reporting for specific materials, systems and methods

We require information from authors about some types of materials, experimental systems and methods used in many studies. Here, indicate whether each material, system or method listed is relevant to your study. If you are not sure if a list item applies to your research, read the appropriate section before selecting a response.

Materials & experimental systems

n/a	Involved in the study	
☐	☒	Antibodies
☐	☒	Eukaryotic cell lines
☒	☐	Palaeontology and archaeology
☒	☐	Animals and other organisms
☒	☐	Clinical data
☒	☐	Dual use research of concern
☒	☐	Plants

Methods

n/a	Involved in the study	
☒	☐	ChIP-seq
☒	☐	Flow cytometry
☒	☐	MRI-based neuroimaging

Antibodies

Antibodies used	Vinculin (Abcam ab18058), Actin (Abcam ab3280), GLS (Abcam ab156876), GAC (RheaBiotech IM-0322), KGA (RheaBiotech IM-0519), HuR (Molecular Probes mp21277 and Cell Signaling 12582), and V5 (Life Technologies 46-1157)
Validation	Data for antibody validation is within the paper, particularly knockdown experiments.

Eukaryotic cell lines

Policy information about cell lines and Sex and Gender in Research

Cell line source(s)	American Type Culture Collection, ATCC
Authentication	Cell lines were not authenticated, with exception to BT549, which was confirmed by "Instituto do Câncer do Estado de São Paulo"
Mycoplasma contamination	Mycoplasma testing was routinely performed by PCR using the primers sequences GPO-3 (5'-GGGAGCAAACAGGATTAGATACCCT-3') and MGSO (5'-TGCACCATCTGTCACTCTGTTACCCTC-3') which amplifies the rRNA of 16 different mycoplasma species. Positive cell cultures were discarded or treated with Plasmocin (InvivoGen). All the cellular assays were performed one to three times.
Commonly misidentified lines (See ICLAC register)	From ICLAC registry, we used HEK293 and BALB/3T3 A31. However, those cells were purchased from ATCC within our institute.

Plants

Seed stocks	n/a
Novel plant genotypes	n/a
Authentication	n/a
